# Dynamic Regulatory Network Reconstruction for Alzheimer's Disease Based on Matrix Decomposition Techniques

**DOI:** 10.1155/2014/891761

**Published:** 2014-06-15

**Authors:** Wei Kong, Xiaoyang Mou, Xing Zhi, Xin Zhang, Yang Yang

**Affiliations:** ^1^Information Engineering College, Shanghai Maritime University, Shanghai 201306, China; ^2^DNJ Pharma and Rowan University, NJ 08028, USA; ^3^Department of Computer Science and Engineering, Shanghai Jiao Tong University, Shanghai 200240, China

## Abstract

Alzheimer's disease (AD) is the most common form of dementia and leads to irreversible neurodegenerative damage of the brain. Finding the dynamic responses of genes, signaling proteins, transcription factor (TF) activities, and regulatory networks of the progressively deteriorative progress of AD would represent a significant advance in discovering the pathogenesis of AD. However, the high throughput technologies of measuring TF activities are not yet available on a genome-wide scale. In this study, based on DNA microarray gene expression data and a priori information of TFs, network component analysis (NCA) algorithm is applied to determining the TF activities and regulatory influences on TGs of incipient, moderate, and severe AD. Based on that, the dynamical gene regulatory networks of the deteriorative courses of AD were reconstructed. To select significant genes which are differentially expressed in different courses of AD, independent component analysis (ICA), which is better than the traditional clustering methods and can successfully group one gene in different meaningful biological processes, was used. The molecular biological analysis showed that the changes of TF activities and interactions of signaling proteins in mitosis, cell cycle, immune response, and inflammation play an important role in the deterioration of AD.

## 1. Introduction

Alzheimer's disease (AD) is a neurodegenerative disease with an insidious onset and progressive stage that inevitably leads to death. The disease progression of AD is slow, and it may take several years from onset of cognitive decline to diagnosis. Although several hypotheses have been proposed and many putative AD susceptibility genes have been witnessed in the past decades, the genetics mechanism and pathogenesis of AD are still unclear. Discovering the changes of gene expressions, transcriptional factors (TFs), and the transcriptional regulatory mechanism, which maps out the coordinated dynamic response of TFs and TGs, would provide a significant advance in genome-wide analysis of AD.

The characteristic pathology change in AD is fibrin deposition in cerebral cortex, and it is the deposition of beta-amyloid (A*β*) in cell space and poly-Tau protein in cell. In pathomorphism, the expressions are senile plaques (SP), neurofibrillary tangles (NFT), cerebrovascular amyloid, dystrophic neuritis, and loss of synaptic connections. Neuroinflammation, as well as dysregulation of lipid metabolism and mitochondrial dysfunction, can also be observed [1]. Currently, the main-stream theory regarding the disease mechanism is the amyloid cascade hypothesis [2]. Recent fast development of high throughput technologies such as DNA microarray technology and statistically computational tools [3] enables large-scale measurements of biological signals to discover critical genes, coregulated gene groups, and transcriptional regulatory network for AD. Ray et al. found 18 signaling proteins, which can efficiently classify AD and control subjects, in blood. Biological analysis of the 18 proteins points to systemic dysregulation of hematopoiesis, immune responses, apoptosis, and neuronal support in presymptomatic AD [4]. Ray et al. identified 6 coexpressed gene modules, each of which represented a biological process perturbed in AD [5]. By combining array analysis and quantitative trait loci (QTL) mapping to characterize the genetic variation and genetic regulatory network, Wang et al. identified many AD-related genes coregulating with App including Gsk3b, Falz, Mef2a, Tlk2, Rtn, and Prkca [6]. Zhang et al. found regulators of tmem59 and reconstructed gene regulatory networks of mouse neural stem cells. 16 out of 36 predicted genes, including Ace, aqp1, arrdc3, cd14, cd59a, cds1, cldn1, cox8b, defb11, folr1, gdi2, mmp3, mgp, myrip, Ripk4, rnd3, and sncg in their constructed network, have been reported to be AD related [7].

Furthermore, to overcome the underlying shortcomings of microarray technology such as small sample size, measurement error, and information insufficiency, some other high throughput technologies, such as protein-protein interaction (PPI), transcriptional factors (TFs), and microRNA knowledge, have been integrated and become more informative and powerful for discovering AD's mechanism. Liang et al. integrated gene expression profiles with prior protein-protein interaction (PPI) network information to reveal significantly perturbed subnetwork in 6 brain regions [8]. Panigrahi and Singh identified novel gene variants in various biological processes, new information for network motif, groups of transcriptional factors (TFs), and miRNA targets to demonstrate that there are extensive links between AD and ageing (AG) [9].

As a progressively neurodegenerative disease in the brain, finding out what kinds of genes crucially change and how TFs dynamically regulate their TGs in the degenerative progress of AD would provide a great achievement in discovering pathogenesis of AD. Since the high throughput technologies of measuring TF activities on the genome-scale are much more limited, the challenge now is to construct the dynamic transcriptional regulatory networks with the help of statistically computational tools. In our study, network component analysis (NCA), a method for determining both the activities and regulatory influence on a set of TFs with their known TGs [10], has been used on degenerative progress of AD samples from incipient to moderate and severe AD. NCA models the expression of a gene as a linear combination of the activities of TFs and regulatory strength from a set of gene expression data and a priori given connectivity information between TFs and their target genes. It has been successfully applied to identifying previously unnoticed oscillatory activity patterns in the yeast cell cycle [10], building network to lipopolysaccharide (LPS) in humans [11], generating a predicted activation time stage of catabolite repressor protein in* Escherichia coli* [12], predicting activities of important transcription factors in a mouse knockout model of human glycerol kinase deficiency, and so on [13–17].

Two inputs: gene expression profiles and a predefined regulatory influence matrix which qualitatively provides the initial estimates of the influence of each TF on the TGs, are required by NCA model. In our study, independent component analysis (ICA) is adopted to extract significant genes from biologically meaningful patterns from different stages of AD gene expression data. Compared with the traditional clustering methods, such as *k*-mean, self-organizing maps (SOM), and hierarchical clustering, which can group each gene in only one class based on the global similarities of the expression profiles, ICA is a biclustering method which can successfully group one gene in different meaningful biological processes and has been successfully applied to microarray data for feature extraction [18, 19], clustering and the classification on yeast cells' cycle [20], and cancer data such as ovarian cancer [21], breast cancer [22], endometrial cancer [23], colon and prostate cancer [24, 25], and acute myeloid leukemia [26]. By combining a priori information between TFs and TGs with significant genes selected by ICA, the selected gene expression profiles and the predefined regulatory influence matrix can be prepared. Finally, we calculate the activities of 10 TFs and regulatory influence on 34 TGs with 71 interactions, using NCA for control, incipient, moderate, and severe AD gene expression data. The dynamic regulatory networks were then successfully built for degenerative progress of AD. Molecular biological analysis demonstrated that the changes of TF activities and their influence on TGs played an important role in AD's onset and deterioration, and the results added additional insights into discovering pathogenesis of AD.

## 2. Methods

### 2.1. Independent Component Analysis

Let the *n* × *m* matrix **X** denote the microarray gene expression data with *m* genes under *n* samples or conditions. *x*_*ij*_ in **X** is the expression level of the *j*th gene in the *i*th sample. In gene expression datasets, the number of genes *m* is much larger than that of the samples *n*, *m* ≫ *n*. Suppose that the data have been preprocessed and normalized; that is, each sample has zero mean and standard deviation, and then the ICA model for gene expression data can be expressed as

(1) $$\begin{array}{c} X=AS. \end{array}$$

In the ICA model of microarray data, the columns of **A** = [*a*_1_, *a*_2_, …, *a*_*n*_] are the *n* × *n* latent vectors of the gene microarray data, **S** denotes the *n* × *m* gene signature matrix or expression mode, in which, the rows of **S** are statistically independent on each other, and the gene profiles in **X** are considered to be linear mixture of statistically independent components **S** combined by an unknown mixing matrix **A**. To obtain **S** and **A**, the demixing model can be expressed as

(2) $$\begin{array}{c} Y=WX, \end{array}$$

where **W** is an *n* × *n* demixing matrix.

The gene expression data provided by microarray technology are considered linear combination of some independent components of specific biological interpretations. The *n*th row matrix **A** contained the weights with which the expression levels of the *m* genes contribute to the *n*th observed expression profile. Therefore, the assignment for the observed expression profiles with different classes is valid for the rows of **A**, and each column of **A** can be associated with one specific expression mode. Since the *n*th column of **A** contains the weights with which *s*_*n*_ contributes to all observations, this column should show large or small entries according to the class labels. After such characteristically latent variables have been obtained, the corresponding elementary modes can be identified to yield useful information for classification. In addition, the distribution of gene expression levels generally features a small number of significantly overexpressed or underexpressed genes that form very biologically coherent groups and may be interpreted in terms of regulatory pathways.

### 2.2. Network Component Analysis

NCA is a tool for analyzing gene expression data of dynamic transcriptional networks. It models the expression of a gene as a linear combination of the activity of each TF that controls the expression of the gene [10]. NCA uses transcription network connectivity to deduce transcription factor activities (TFAs) and TF-gene regulation control strengths (CS) from gene expression data. The following transcription regulation model is used:

(3) $$\begin{array}{c} \frac{E_{i}\left(t\right)}{E_{i}\left(0\right)}=\prod {\left(\frac{{\text{TFA}}_{j}(t)}{{\text{TFA}}_{j}(0)}\right)}^{{\text{CS}}_{ij}}, \end{array}$$

where *E*_*i*_(*t*) is the expression level of gene *i*, TFA_*j*_(*t*), *j* = 1, …, *L*, is the activity level of TF *j*, CS_*ij*_ represents the control strength of TF *j* on gene *i*, and (*t*) and (0) designate condition *t* and reference condition 0. Log-linear transformation as a standard tool is used to approximate this nonlinear system. The matrix form of (3) after taking the logarithm is shown as follows:

(4) $$\begin{array}{c} \left[E\right]=\left[C\right]\left[P\right]+\Gamma . \end{array}$$

Here [**E**]  (*N* × *M*) is a gene expression matrix of *N* genes in *M* samples, matrix [**C**]  (*N* × *L*) represents the connectivity strength of each transcriptional factor (TF) *j* on target gene *i*, matrix [**P**]  (*L* × *M*) denotes TFAs on *M* samples, *N* is the number of genes, *L* is the numbers of TFs, *M* is the number of experiments, and Γ is the residual of the model. The element *c*_*ij*_ in matrix [**C**] is set to 0 if there is no evidence to suggest regulation of gene *i* by TF_*j*_; otherwise, it is set to a nonzero number as an initial value.

As the decomposition of [**E**] into component matrices is inherently nonunique, Liao et al. proved that if [**C**] and [**P**] satisfy the uniqueness criteria, NCA can guarantee a unique solution up to a scaling factor for any given residual Γ. This criterion clearly links NCA results to the biological system and makes interpretation straightforward.

To find the best solution of (4), the least-square algorithm is performed:

(5) min⁡ ||[E]−[C][P]||2,s.t.      C∈Z0,

where **Z**_0_ is the topology induced by the network connectivity pattern. Then the actual estimation of [**C**] and [**P**] is performed by a two-step alternating least-squares algorithm which exploits the biconvexity properties of linear decompositions. The least-square constrain is equivalent to a maximum-likelihood procedure in the presence of Gaussian noise with independent and identically distributed component. For details see [10] by Liao et al. (2003).

To perform NCA, two inputs are needed. One is gene expression profiles of TGs, matrix [**E**]. The other is matrix [**C**_0_], a predefined regulatory influence matrix which provides the initial estimates of the influence of each TF on its TGs. The original biologically qualitative regulatory influence of TFs together with a set of known TGs is obtained from the TRANSFAC public dataset of BIOBASE (http://www.gene-regulation.com). There are 67375 regulatory interactions including 1972 TFs and 6561 TGs without overlap in this dataset. To define key TFs and regulatory network of AD, this TF-TG regulatory interaction dataset is matched with the significant genes extracted by ICA and differentially expressed in all incipient, moderate, and severe AD gene datasets. 10 TFs with their TGs and in total 71 interactions are finally chosen for NCA model. Then, matrix [**E**] is the gene expression of the TGs in different stages of AD samples, and the initial value of matrix [**C**_0_] is set to 1 if there is connectivity strength of TG_*i*_ by TF_*j*_; otherwise, it is set to 0.

## 3. Results and Discussion

### 3.1. ICA Results

To evaluate ICA applied to AD DNA gene expression data, we use the dataset of hippocampal gene expression of control and AD samples from GEO DataSets, series GSE1297, offered by Blalock et al. [27]. The hippocampal specimens they used are obtained through the Brain Bank of the Alzheimer's Disease Research Center at the University of Kentucky. The human Gene Chips (HG-U133A) of Affymetrix and Microarray Suite 5 are used to analyze the microarray data. The procedures for total RNA isolation, labeling, and microarray are described in [27, 28]. There are in total 9 control, 7 incipient, 8 moderate, and 7 severe AD samples included in this dataset with 22283 gene expressions in each sample.

According to Kaissi and his colleagues [3], significance analysis of microarrays (SAM) is better than fold change (FC) and *t*-test in terms of sensitivity and specificity. In our study, to reduce significant noises, SAM was applied to all 31 samples as a preprocessing method. Three groups were built for ICA feature gene extraction, respectively: control-incipient AD (C-I), control-moderate AD (C-M), and control-severe AD (C-S) samples. After SAM preprocessing, around 4500–5000 genes were reserved for each group. FastICA, presented by Hyvärinen and Oja [29], as the fastest ICA method, was applied to 3 groups to discover significant genes between control and different courses of AD, respectively.

In FastICA algorithm, nonlinear function *g*(*u*) = tanh(*a*1∗*u*), where *a*1 was a constant, was used as the probability density distribution of the outputs *u* during the iteration. As the FastICA algorithm relied on random initializations for its maximization and had the problem of convergence to local optima, we iterated FastICA 50 times to alleviate the instability of the slightly different results in each iteration. For each IC in each time, significant genes were not the same, and we selected top hundreds of genes as significant genes by calculating the number of times for 50 times. FastICA identified 245, 268, and 324 significant genes for C-I, C-M, and C-S group, respectively. By integrating the genes of these 3 different stages of AD and excluding overlapping part, we finally extracted 740 genes as significant genes for the whole AD dataset.

From the biological analysis of FastICA results we found that the significant genes and their relevant pathways were related in immunoreactions, metal protein, membrane protein, lipoprotein, neuropeptide, cytoskeleton protein, binding protein, and ribosomal protein, playing prominent roles in AD and connecting the activation patterns with AD phenotypes. FastICA also found that many oncogenes and phosphoric proteins were significantly lowly expressed in all the 3 courses of AD.

To be compared with ICA results, here we present our previous experimental results of nonnegative matrix factorization (NMF) to analyze the efficiency of different biclustering methods on AD gene expression data [30]. NMF is a matrix factorization method, which assumes that the given gene expression data are combined of positive metagenes with meaningful local biological representation, and represents the original data as a linear combination into reduced sets based on nonnegative constrain. By performing NMF, more than 1500 significant genes were extracted. Lots of them were related to metal metabolism and inflammation; for instance, many upregulated genes were found to be in conjunction with zinc and calcium (Ca (2+)). Furthermore, NMF also identified the genes related to cell growth, cell cycle, apoptosis, cellular fission, and cell repair. The shortcomings of NMF method were that the number of significant genes was hard to reduce, and the method focused on meaningful biological processes due to the unapparent difference among the metagenes.

Another matrix decomposition method and principle component analysis (PCA) on the control and severe samples of the same AD microarray dataset in our previous research [31] were presented here. In the experiment, control samples were modeled by PCA and represented each gene as a linear combination of the dominant principal components (PCs). Then the severe AD samples were projected on the PCA model and the scores can be extracted. The 100 most different genes between the scores obtained by control data and severe AD data were selected for further biological analysis. The results showed that they contained genes in immunoreactions, metal protein, membrane protein, lipoprotein, neuropeptide, cytoskeleton protein, binding protein, ribosomal protein, and phosphoric protein. The limitation of PCA model is that the number of significant genes, which can be extracted, is much fewer than that of ICA.

In summary, the molecular biological analysis of the significant gene extraction confirmed the added value of ICA over NMF and PCA methods in identifying known and novel genes in biological processes. Our results indicate that ICA enables researchers to extract potentially relevant gene expression information from microarray gene expression data and map closer to AD pathways. Moreover, all these 3 methods are based purely on statistical constraints and they do not use any biological knowledge or any transcriptional regulatory structures; therefore, their results cannot contain biologically transcriptional regulatory networks.

### 3.2. NCA Results

The second stage in this study was to find the activities of TFs and regulatory influence on TGs for 3 courses of AD by NCA. We used the biologically qualitative regulatory influence of TFs and genes from the TRANSFAC public dataset of BIOBASE (http://www.gene-regulation.com) to find the TF genes in ICA results. 23 TFs with their TGs were found in the selected significant genes. Then top 10 TFs, regulating TGs with the number exceeding 13, were selected. These 10 TFs with 34 TGs without overlap and 71 interactions were provided for NCA model.  Table 1 shows these 10 TFs with their TGs, chromosome locations, and theirpromoter ID, which will add further information to discovering pathogenesis of AD. Two inputs were prepared for NCA model: one was matrix [**E**], which presented the gene expression profiles of TGs provided by the original gene expression data of AD, and the other was predefined initial matrix [**C**_0_] which reflected the relative contribution of the TFs on TGs. Then NCA was applied to control, incipient, moderate, and severe AD datasets, respectively. Take the incipient AD samples as an example, in which [**E**] was a 34 × 7 matrix which denoted the microarray expression profiles of 34 TGs in 7 incipient AD samples. Matrix [**C**_0_] (34 × 10) represented the predefined connectivity strength by the 71 interactions obtained above, and the element *c*_*ij*_ in matrix [**C**_0_] was set to 0 if there was no connectivity strength of TG *i* by TF_*j*_; otherwise, it was set to 1 as an initial value. Matrix [**P**] (10 × 7) contained the TFAs of 10 TFs on 7 samples. After performing NCA, the TF activities (matrix [**P**]) and the control strength (matrix [**C**]) were quantitatively obtained for different courses of AD.

**Figure 1:**
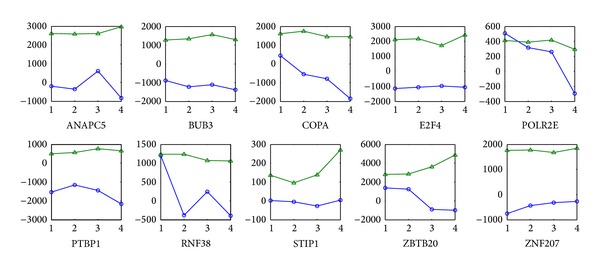
10 transcription factor activities (TFAs) compared to their gene expression profiles. Blue solid lines with “○” and green solid lines with triangle denote TFAs and their gene expression profiles, respectively, numbers 1~4 on *x*-axis denote 4 stages of control, incipient, moderate, and severe AD, and *y*-axis is the activities or gene expression values of TF.

Transcription of genes is controlled by a small number of TFs whose activation via posttranslational modification or ligand binding is the determining factor of gene expression. In general, TFAs are not always correlated with their gene expression profiles.  Figure 1 gives the comparison of the estimated TFAs and their gene expression profiles in microarray gene expression profiles. Each subfigure shows the activities and gene expression of TF in control, incipient, moderate, and severe AD. We found that most of TFs represent a novel approach to control cellular processes.  Figure 1 showed that the activities of COPA, POLR2E, and ZBTB20 were gradually depressed, which meant that they were deactivated over the deterioration of AD. E2F4, as well as ZNF207, was activated within the deterioration of AD. Moreover, some TFAs were neither monotonic decreasing nor increasing. For example, ANAPC5, BUB3, and RNF38 were deactivated in incipient AD, then greatly activated in moderate AD, and finally decreased in severe AD. PTBP1 was activated in incipient AD and repressed in moderate and severe AD gradually. STIP1 was repressed in incipient and moderate AD and then activated in severe AD. The molecular biological analysis of the functions of TFs and TGs in AD was discussed in the next section.

### 3.3. Dynamic Regulatory Network of AD

**Figure 2:**
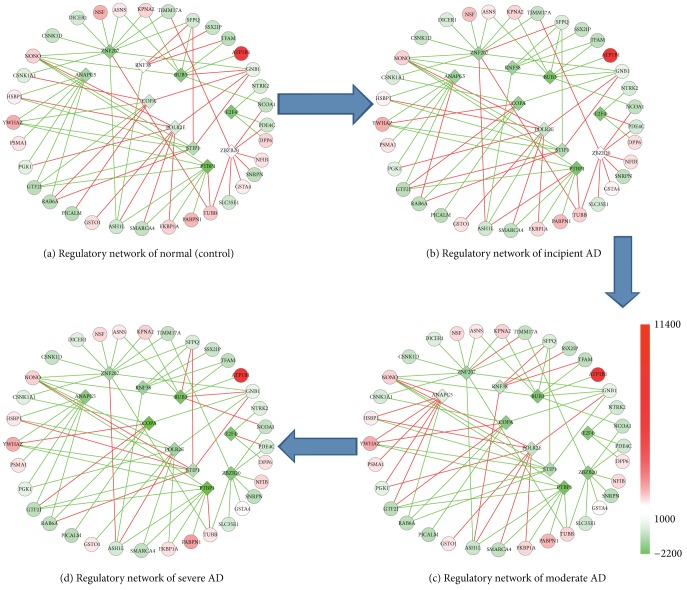
Dynamic transcriptional regulatory network for AD. Transcriptional regulatory network of (a) control, (b) incipient, (c) moderate, and (d) severe AD samples. Diamond in the middle denoted TFs with activity values in different colors, circles with different colors presented the gene expression of target genes, and the lines between TFs and target genes with different colors noted the control strength.

Based on the quantitative results of TFAs (matrix [**P**]), control strength on the TGs (matrix [**C**]), and the original microarray gene expression, our original goal of reconstructing the dynamic regulatory networks for different courses of AD was done in Figure 2. 10 TFs were noted with diamond in the middle of each course, and target genes which were activated or repressed by TFs were noted in circles with control strength lines. According to Figure 2, we discovered many additional insights in biological regulatory pathways of different courses of AD. For example, ANAPC5, which was greatly activated in the moderate AD, is a subunit of APC, and APC is essential for cells to progress through anaphase, exit from mitosis, and prevent a premature entry into S phase [32].

BUB3 was deactivated in incipient AD. It is a crucial component in the formation of the mitotic spindle assembly complex, which forms a complex with other important proteins [33]. ZNF207, which is subunit of ZNF, was activated in the whole AD. Zinc finger transcription factors (ZNF) are transcription factors composed of a zinc finger binding domain and any of a variety of transcription-factor effector domains which exert their modulatory effect in the vicinity of any sequence to which the protein domain binds modulating gene expression directly at the DNA level to be engineered to target virtually any gene [34]. Reference [35] showed that ZBTB20 can be regulated by ZNF207. In our experiment, it showed that some TFs were related to cell cycle and regulated the genes of cell cycle obviously; for example, ANAPC5, BUB3, and ZBTB20 regulated gene expression of CEP27. The CEP family protein is the active component of centrosome and plays a vital role in centriole biogenesis and cell cycle progression control [36]. Furthermore, ZBTB20 could inhibit I*κ*B*α* gene transcription, govern I*κ*B*α* protein expression, and then promote NF-*κ*B activation [37].

G3BP1 mediated effect on proliferation in lung cancer and breast cancer [38, 39]. Interestingly, in AD research, our data showed G3BP1 was regulated by ANAPC5, COPA, PTBP1, STIP1, and ZNF206. The result suggested that G3BP1 had biological significance in AD process. Our data also exhibited that the stress-inducible protein-1 (STPI1) regulated HSP90AB1 and HSPD1. STIP1 is an adaptor protein that coordinates the functions of HSP70 and HSP90 in protein folding. It is thought to assist in the transfer of proteins from HSP70 to HSP90 by binding both HSP90 and substrate-bound HSP70. STPI1 was upregulated in the ischemic brains from humans and rodents. The increase in STPI1 expression in vivo was not cell-type specific, as it was found in neurons, glia, and endothelial cells [40]. STIP1 also stimulates ATPase activity of HSP70 and inhibits ATPase, activity of HSP90, which suggests that it regulates both the conformations and ATPase cycles of these chaperones [41–43].

COPA coatomer is a cytosolic protein complex that binds to dilysine motifs and reversibly associates with Golgi non-clathrin-coated vesicles, which further mediates biosynthetic protein transport from endoplasmic reticulum (ER) via the Golgi up to the trans-Golgi network [44]. From Figure 1 we can see that the activities of COPA were progressively inhibited in the whole course of AD.

TFII (GTF2I) was classified as a general transcription factor when it was first identified. TFII-I (GTF2I) identified and validated novel neuronal targets which affected the PI3 K and TGF*β* signaling pathways in vivo, and it played a major role in the neurodevelopmental features of Williams-Beuren syndrome (WBS) [45]. TFII (GTF2I) was the main cause of the neurocognitive profile [46]. Our results exhibited that, in normal (control) stage, COPA, POLR2E, and STIP1 negatively regulated TFII (GTF2I). In other 3 stages, COPA, POLR2E, and STIP1 positively regulated TFII (GTF2I). In incipient AD, COPA and STIP1 positively regulated TFII (GTF2I); in moderate AD, ANAPC5 and COPA positively regulated TFII (GTF2I); in severe AD, only COPA positively regulated TFII (GTF2I), but STIP1 and PTBP1 negatively regulated TFII (GTF2I). It hints that, in different courses of AD, the TFs play a different role in regulating even the same TGs.

From the original gene expression profiles we can see that ZFP207, as a TF, was increased gradually in the process of the deterioration of AD; by contrast, the gene expression of BUB3, COPA, POLR2E, and ZBZB20 was decreased gradually. The target Gene NONO (p54nrb) protein is a RNA-binding protein, and the gene that interacted with RNA could be selectively modulated by phosphorylation during mitosis [47]. As Table 1 showed, NONO (p54nrb) can be regulated by many kinds of TFs, such as ZNF207, BUB3, PTBP1, POLR2E, STIP1, and ANAPC5. Many of them were related to mitosis and cell growth. ZNF207 (BuGZ) is a gene associated with spindle microtubules and it can regulate chromosome alignment. ZNF207 (BuGZ) directly binds and stabilizes Bub3 and uses its microtubule-binding domain to enhance the loading of Bub3 to kinetochores that have assumed initial interactions with microtubules in prometaphase [48].

Moreover, many target genes in our dynamic regulatory networks, such as CK1alphaLS, CLIP1, TUBB, ENO1, CSNK1D, BUB3 CSNK1A1, and SFPQ, were also related to cell growth [49, 50]. Our data indicated that SFPQ was regulated by ZNF207, BUB3, STIP1, and RNF38. SFPQ pathology may progress together with the tau pathology in AD [51]. ENO1 was regulated by ZNF207, BUB3, PTBP1, E2F4, POLR2E, and STIP1. The gene ENO1 was related to cell growth and migration [52]. Furthermore, as a neuronal expressed tubulin gene, the expression profiles of TUBB which is associated with a spectrum of disorders affecting cerebral cortex formation were decreased gradually in deteriorative processes of AD [50]. Our dynamic regulatory network showed that many TFs and TGs were closely related to the mitosis and cell growth. The results illustrated that regulating the genes related to mitosis and cell growth would become an important way for AD treatment.

Recently, some similar studies on AD gene expression data have been proposed and obtained some meaningful results. For example, Panigrahi and Singh applied an integrative systems biology approach to AD pathway and regulatory network analysis [9]. They identified 26 novel genes and variants associated with AD and ageing and some coexpression networks. They grouped TFs according to the TFBS classes into 10 groups and illustrated that they were related to immune system, DNA binding domain, central nervous system, and so on. Furthermore, they also found some novel information of network motifs and unique miRNA targets as a regulatory process for AD. By contrast, our studies focused on finding the dynamic responses of genes, signaling proteins, and activities of TFs on TGs in the progressively degenerative progress of AD, which would represent a deep understanding of the underlying transcription regulatory process for pathogenesis of AD.

The characteristic pathological change in the deteriorative process of AD is that the cerebral cortex begins to shrink as more and more neurons stop working and die. This phenomenon with the growth of neurons to repair and cell growth has close relation to the environment. In our results, some causes of AD were discovered by the comprehensively biological analysis of the dynamical changes of TFs on TGs, which were related to mitosis, cell growth, immune response, and inflammation. And our future study will also focus on the mitosis, cell growth, immune response, and inflammation of AD to find its real pathogenesis.

## 4. Conclusions

Microarray technologies enable the simultaneous measurement of all mRNA transcripts and therefore make it possible to reconstruct the gene regulatory network. The traditional models for gene network analysis such as ICA did not use any transcription regulatory information and were purely based on mathematic and statistical properties of the regulatory signals; thus they cannot correctly reconstruct the regulatory network. In this study, by combining biochemical information, that is, the connective strength between TFs and their regulated genes with gene expression profiles, NCA was utilized to deduce the dynamic regulatory network of AD. ICA was firstly applied to identifying significant genes from various biological processes of different courses of AD. Then NCA was applied to inferring regulatory actions of TF activities from gene microarray profiles and partial TF-TG connectivity information.

From the molecular biological analysis of the reconstructed dynamic regulatory network of AD, we found some transcriptional regulatory pattern in more biological insight for AD. We found that some TFs were activated during the whole AD deterioration or activated in the beginning of AD, such as ANAPC5, E2F4, PTBP, and ZNF207, and were proved to play an important role in centriole biogenesis and cell cycle progression control. Another gene, GTF2I, which was important in neurodevelopment and neurocognition, was found dynamically regulated from normal to AD stage. Some TFs, such as COPA, PTBP1 and STIP1, were inhibited in AD. Gene G3BP1 coregulated by these TFs was found low expressed in binding protein in AD. However it always proliferated in cancers. It hints that some biological relationship or signal regulatory pathway should be coordinated between AD and cancer. In general, the dynamically characteristic analysis of TFs on TGs of deteriorative courses of AD helps us focus the future study on mitosis, cell growth, immune response, and inflammatory reaction for the pathogenesis of AD.

## Figures and Tables

**Table 1:** Selected TFs and the target genes.

Selected TFs	Description	Chromosome locations and promoter ID	Target genes	Number of TGs
ZNF207	Zinc finger protein 207	17q12, promoter ID: 17074	GNB1, CLIP1, SFRS18, CSNK1D, SFPQ, CSNK1A1, ASNS, ZBTB20, TUBB, TM2D1, TIMM17A, ENO1, ASH1L, NONO, KPNA2, REEP5, NSF, HSPD1, UBE2D3, DICER1, and G3BP1	21

BUB3	BUB3 budding uninhibited by benzimidazoles 3 homolog	10q26 promoter ID: 5046	TARDBP, ATP1B1, SFPQ, GNB1, CLIP1, SET, ARPC5L, NCOA1, NOL5A, PPIA, CEP27, REEP5, ENO1, ASNS, ZBTB20, HSPD1, PSMB1, NONO, and KPNA2	19

PTBP1	Polypyrimidine tract binding protein 1	19p13.3 promoter ID: 20018	PABPN1, UBL3, REEP5, KIAA0232, SET, G3BP1, ENO1, GTF2I, SMARCA4, ASH1L, WDR1, CSNK1D, HSP90AB1, TUBB, FKBP1A, YWHAZ, HSPD1, VAMP4, and NONO	19

ZBTB20	Zinc finger and BTB domain containing 20	3q13.2 promoter ID: 119577	POGZ, NFIB, CIRBP, TIMM17A, CEP27, SNRPN, ARHGAP5, TUBB, RUFY3, EZH1, NCOA1, GSTA4, KTN1, RPL35A, GPRASP1, PDE4C, SLC35E1, RABGEF1, and ZNF160	19

COPA	Coatomer protein complex, subunit alpha	1q23–25 promoter ID: 2753	G3BP1, TM2D1, GNB1, GTF2I, RIPK5, DCTN4, SCAMP1, HSPA8, RAB6A, CANX, WDR1, ACTB, CSNK1A1, HSP90AB1, and YWHAZ	15

E2F4	E2F transcription factor 4, p107/p130 binding	16q21-q22 promoter ID: 15321	RUFY3, NTRK2, GABRB2, ENO1, BAIAP2, KTN1, DPP6, PDE4C, EZH1, ITFG1, NECAP1, JAK1, NLRP1, and CHERP	14

POLR2E	Polymerase (RNA) II (DNA directed) polypeptide E, 25 kDa	19p13.3 promoter ID: 22868	SMARCA4, ASH1L, RIPK5, HSBP1, UBE2D3, GNB1, HSPA8, NONO, GSTO1, PGK1, ENO1, WDR1, FKBP1A, and HSP90AB1	14

STIP1	Stress-induced phosphoprotein 1	11q13 promoter ID: 6654	SFPQ, HSBP1, GTF2I, FAM108B1, GNB1, G3BP1, RAB6A, WDR1, HSPA8, ENO1, NONO, YWHAZ, HSPD1, and HSP90AB1	14

ANAPC5	Anaphase promoting complex subunit 5	12q24.31 promoter ID: 9688	CEP27, FAM108B1, G3BP1, GTF2I, HSBP1, HSP90AB1, HSPA8, NONO, PGK1, PSMA1, RAB6A, RIPK5, and YWHAZ	13

RNF38	Ring finger protein 38	9p13-12 promoter ID: 42818	REEP5, SSX2IP, DCTN4, DCUN1D4, HSPA8, C1orf43, SFPQ, ASH1L, ITFG1, HSP90AB1, YWHAZ, MGEA5, and FOXJ3	13
